# Endogenous Sex Hormones Explain Variation in Long-Term SARS-CoV-2 Antibody Persistence Beyond Biological Sex

**DOI:** 10.3390/antib15040072

**Published:** 2026-08-07

**Authors:** Nicole Ivaska, Laura Rothschild

**Affiliations:** Department of Health Sciences, Ursinus College, Collegeville, PA 19426, USA; laroth@upenn.edu

**Keywords:** antibodies, SARS-CoV-2, COVID-19, sex hormones, estradiol, testosterone, human

## Abstract

**Background**: Sex-related differences in SARS-CoV-2 viral susceptibility, severity, and recovery are prevalent. Factors such as age and biological variation provide some explanation for immune response, yet the use of biological sex as a variable may limit understanding of underlying individual mechanisms related to endocrine–immune function involved in long-term immune memory. Endogenous sex hormone concentrations provide a means to assess the magnitude or durability of antibody persistence and disease recovery. **Objective**: This study examined salivary concentrations of 17β-estradiol and testosterone, as well as the number of weeks since prior SARS-CoV-2 viral infection. The study aimed to determine whether endogenous sex hormones contributed additional explanatory value to antibody persistence beyond biological sex. **Methods**: A cross-sectional study included 75 college students (63% female) who reported weeks since previous SARS-CoV-2 infection (M = 15.41, SD = 7.13), confirmed through a positive SARS-CoV-2 anti-N rapid antibody test. Participants provided saliva samples, which were tested for concentration of 17β-estradiol and testosterone. Hierarchical multiple regression analyses examined the effects of biological sex and endogenous sex hormones on the persistence of SARS-CoV-2 antibodies following natural infection. **Results**: 17β-estradiol was the strongest positive predictor of SARS-CoV-2 antibody persistence (*β* = 0.63, *p* < 0.001). Departing from previous findings, biological sex was not a significant predictor of antibodies targeting the SARS-CoV-2 N protein. **Conclusions**: Findings underscored the role of estradiol on immune recovery in a healthy young adult population. Individual hormone variation appears more informative than sex classification for understanding antibody persistence and immune response.

## 1. Introduction

The SARS-CoV-2 pandemic highlighted individual variability in susceptibility to infection, disease severity, and the durability of immune responses following infection. Studies have reported sex-related differences in COVID-19 health outcomes, including disease progression, pre-existing health burden, and vaccine response [1,2]. While natural infection stimulates adaptive responses to establish virus-specific immune memory [3,4], considerable variation exists in the magnitude and durability (noted by rate of decay) of these responses [5].

The major structural components of the SARS-CoV-2 virus include the spike (S), nucleocapsid (N), membrane (M), and envelope (E) proteins, each of which contributes to viral replication, assembly, or host immune recognition [6]. Following SARS-CoV-2 infection, the adaptive immune system produces highly specialized cellular and humoral responses such as virus-specific antibodies, including immunoglobulin M (IgM), immunoglobulin A (IgA), and immunoglobulin G (IgG). Anti-S antibodies are produced following both natural SARS-CoV-2 infection and most COVID-19 vaccination [7,8]. Anti-N antibodies distinguish a serological biomarker of prior natural SARS-CoV-2 infection since most COVID-19 vaccines authorized in the United States encode only the viral spike (S) protein, while whole-virus vaccines may also elicit anti-N responses [7,8].

Although circulating antibody concentration often declines following recovery, memory B-cells and T-cells may persist for extended periods and facilitate a rapid immune response upon subsequent exposure to the virus [9,10]. These mechanisms are central to understanding individual differences in the persistence of SARS-CoV-2 antibodies and long-term immune protection following infection. Given the central role of adaptive immune responses in the generation and maintenance of immunological memory, factors that influence B-cell and T-cell function may contribute to variation in persistence of SARS-CoV-2 antibodies following infection [11,12].

Understanding factors that influence antibody persistence is particularly important given documented sex-based differences in COVID-19 outcomes. Previous studies noted that males experienced higher rates of COVID-related severe disease and mortality than females [5,13], a pattern also observed through meta-analysis of COVID-19 complications [14]. Additionally, evidence suggests that men may exhibit delayed viral clearance compared to women, suggesting potential sex-related differences in antiviral immune recovery [15]. Sex differences in immune response have been attributed to the immunomodulatory effects of sex hormones, particularly 17β-estradiol and testosterone [16,17,18]. Sex steroid hormones play a pivotal role in various physiological processes—cardiac function [19], skeletal strength [20], skeletal muscle metabolism [21], immune cell modulation [18,22], and the development of human secondary sex characteristics. However, biological sex is often the variable of consideration in immune-endocrine interactions despite variation in hormone concentrations within females and males.

Despite growing evidence that sex hormones influence immune function, relatively few studies have examined the relationship between endogenous sex hormones and the persistence of SARS-CoV-2-specific antibodies following natural infection. Many previously reported findings elucidate vaccine-mediated persistence of SARS-CoV-2 anti-S antibody or combined immunological protection of vaccine-mediated anti-S antibodies and anti-N antibodies following natural infection [7,12]. However, understanding SARS-CoV-2 anti-N antibody persistence provides critical determination of the humoral immune response, viral evolution, and mechanisms for long-term pathophysiology and disease sequelae. Consequently, investigation of endogenous sex hormones as potential modulators of post-infection antibody persistence may provide valuable insight into the biological mechanisms underlying variation in long-term immunity following SARS-CoV-2 infection.

Despite evidence that biological sex influences COVID-19 outcomes, the extent to which circulating sex hormones contribute to long-term SARS-CoV-2 antibody persistence remains unclear. Therefore, the aim of the present study was to examine associations between salivary concentrations of 17β-estradiol, testosterone, and SARS-CoV-2 antibody persistence following natural infection. In this study, antibody persistence refers to the weeks elapsed following infection at which anti-N IgG antibodies remained detectable at the time of assessment. Specifically, we sought to determine whether endogenous sex hormones explain variation in SARS-CoV-2 antibody persistence beyond biological sex alone.

## 2. Materials and Methods

### 2.1. Study Design

This cross-sectional study examined associations between endogenous sex hormone concentrations and SARS-CoV-2 antibody persistence following natural infection. Salivary concentrations of 17β-estradiol and testosterone were quantified as continuous variables (pg/mL) using enzyme-linked immunosorbent assays (ELISAs). SARS-CoV-2 anti-N IgG antibodies were assessed qualitatively using the Megna COVID-19 IgG Antibody Test to verify evidence of prior SARS-CoV-2 infection. Antibody persistence was operationalized as the continuous variable representing the number of weeks elapsed between a participant’s self-reported SARS-CoV-2 infection and antibody assessment. Since antibody status was assessed at a single time point, persistence reflects the duration over which antibodies remained detectable from infection date to assessment date rather than longitudinal changes in antibody concentrations.

A power analysis was conducted a priori using G*Power 3.1 for hierarchical multiple regression assessing the contribution of 17β-estradiol and testosterone beyond biological sex. Assuming a medium effect size (*f*^2^ = 0.15), α = 0.05, power = 0.80, two tested predictors, and three total predictors in the final model, a minimum sample size of 68 participants was required. The final sample of 75 participants exceeded this threshold, providing adequate power to detect a medium-sized increase in explained variance associated with circulating sex hormones.

### 2.2. Participants

Participants were recruited from a residential liberal arts college in the northeastern United States between January and April 2023 using campus-wide email announcements and institutional advertising. Consistent with the traditional undergraduate population, participants were expected to be predominantly young adults between 18 and 24 years of age. This relatively narrow age range reduced the likelihood of substantial age-related variation in endogenous sex hormone concentrations, thereby minimizing age as a potential source of hormonal variability within the study cohort. Eligibility criteria included being at least 18 years of age and having experienced a confirmed SARS-CoV-2 infection or received a SARS-CoV-2 vaccination at least two weeks prior to participation. All participants provided written informed consent before enrollment.

### 2.3. Procedure

Participants attended a single laboratory session lasting approximately 30 min. Participants were instructed to abstain from eating, drinking, smoking, or oral hygiene activities for at least one hour prior to testing to minimize factors known to influence salivary biomarkers. Participants completed a demographic questionnaire and comprehensive health history survey that included dates of previous SARS-CoV-2 infection, date of previous SARS-CoV-2 (COVID-19) vaccination, COVID-19 symptom history, concurrent medical diagnoses, current medications, and reproductive health information. Medical history was reviewed for conditions and treatments known to alter endogenous sex hormone concentrations, and no participants reported relevant medical conditions or use of medications expected to substantially affect estradiol or testosterone levels. Female participants using only intrauterine devices were eligible for study inclusion because ovarian function and endogenous sex hormone production are generally preserved with this contraceptive method [23].

Female participants self-reported onset of menstrual cycle, and saliva collection was coordinated during the early follicular phase (cycle days 2–7). This interval was selected because female estradiol concentration is generally at a physiological nadir and exhibits less variability during this phase than during the periovulatory and luteal phases, thereby reducing menstrual cycle-related variability in endogenous estradiol measurement [24]. All saliva samples were collected during a standardized morning time window between 9:00 h and 12:00 h to minimize potential circadian variation in endogenous hormone concentrations. Previous studies have demonstrated significant diurnal variation in estradiol and testosterone concentrations in both women and men, with peak concentrations typically occurring in the morning [24,25].

In total, 75 participants completed the survey and subsequent antibody and hormone samples. The date of last SARS-CoV-2 infection was identified through the survey questions, “Have you had a previous confirmed positive COVID-19 test (either rapid antigen test or PCR test)?” and “If yes, please indicate Month/Year of positive test.” Dates were specifically counted in weeks since the reported SARS-CoV-2 infection until the sample collection day.

### 2.4. Antibody Assessment

Participants completed an iHealth COVID-19 Antigen Rapid Test (iHealth Labs, Inc., Sunnyvale, CA, USA) to screen for active SARS-CoV-2 infection. Participants with a negative SARS-CoV-2 rapid antigen test proceeded with all study procedures. Participants who tested positive had study procedures deferred until at least two weeks had elapsed and a subsequent negative SARS-CoV-2 test was obtained. All participants tested negative on the day of biological sample collection.

SARS-CoV-2 antibodies were assessed using the Megna Health COVID-19 IgM/IgG Rapid Test (Megna Health, Inc., Exton, PA, USA) according to manufacturer instructions. On the collection day, capillary whole blood was collected by fingerstick using a sterile, single-use automatic lancet. Approximately 5 μL of whole blood (one blood drop) was collected using the capillary collection straw and transferred directly to the specimen well of the test card. Two drops of the manufacturer’s sample buffer were added to the designated buffer well, and the assay evaluated the presence or absence of SARS-CoV-2 anti-N antibodies according to the manufacturer’s instructions. Testing procedures were strictly followed to verify current natural infection following self-reported SARS-CoV-2 infection date. Participants were confirmed SARS-CoV-2 seropositive if IgG antibodies were detected from the rapid antibody test and confirmed seronegative if IgG antibodies were not detected from the rapid antibody test.

### 2.5. Hormone Analysis

Participants collected approximately 3 mL of saliva via passive drool into sterile collection tubes. Samples were stored at −20 °C until analysis. Prior to assay, samples were thawed and centrifuged at 1500× *g* for 15 min to remove particulate matter. Salivary concentrations of 17β-estradiol and testosterone were quantified in duplicate using commercially available ELISA kits according to manufacturer protocols (Salimetrics, State College, PA, USA). Absorbance was measured at 450 nm, and hormone concentrations (pg/mL) were calculated from four-parameter logistic standard curves. Intra-assay coefficients of variation below 10% were considered acceptable.

### 2.6. Statistical Analysis Plan

All statistical analyses were conducted using IBM SPSS Statistics Version 29 (IBM Corp., Armonk, NY, USA). Descriptive statistics were calculated for all study variables and examined for normality, outliers, and missing data. Estradiol and testosterone concentrations were standardized into z-scores prior to analysis to improve interpretability of regression coefficients and reduce multicollinearity in interaction models. Pearson correlation analyses were conducted to examine bivariate associations among hormone concentrations and antibody persistence. Hierarchical multiple linear regression was subsequently performed to determine whether circulating sex hormone concentrations explained variation in SARS-CoV-2 antibody persistence beyond biological sex. Antibody persistence, measured as the duration of detectable SARS-CoV-2 antibodies in weeks, served as the dependent variable.

In step 1, biological sex (female = 0, male = 1) was entered as the predictor to establish the baseline association between sex and antibody persistence. In step 2, standardized estradiol and testosterone concentrations were added to the model to assess whether hormone levels explained additional variance in antibody persistence beyond biological sex. Improvement in model fit was evaluated using changes in explained variance (Δ*R*^2^).

In step 3, interaction terms were exploratory and introduced to determine whether the relationships between hormone concentrations and antibody persistence differed by biological sex. Interaction terms were created by multiplying biological sex by the standardized estradiol and testosterone variables (Sex × Estradiol and Sex × Testosterone). The final model therefore included biological sex, standardized 17β-estradiol, standardized testosterone, and both interaction terms. Significant interaction effects were interpreted as evidence that the association between hormone concentrations and antibody persistence varied by sex. Regression assumptions, including linearity, homoscedasticity, normality of residuals, and multicollinearity, were evaluated prior to interpretation. Variance inflation factors (VIF) and tolerance statistics were used to assess multicollinearity. Statistical significance was set to *p* < 0.05.

### 2.7. Ethics Statement

The study protocol was approved by the Ursinus College Institutional Review Board prior to participant recruitment. All procedures were conducted in accordance with the Declaration of Helsinki, and all participants provided written consent before participation.

## 3. Results

Women (63%) comprised most of the sample, with 91% of participants self-reporting an age of 18–24 years. Consistent with Salimetrics manufacturer standards, females (N = 47, M = 52.94, SD = 20.55) exhibited lower testosterone levels than males (N = 28, M = 149.68, SD = 47.88). Additionally, females (N = 47, M = 1.40, SD = 0.57) exhibited similar 17β-estradiol levels as males (N = 28, M = 1.70, SD = 0.73) due to baseline timing of data collection. Females (N = 47, M = 15.49, SD = 7.55) and males (N = 28, M = 15.29, SD = 6.49) reported similar SARS-CoV-2 antibody persistence in weeks since infection. All participants demonstrated SARS-CoV-2 IgG anti-N antibody on rapid test kits. Additionally, all participants received a COVID-19 vaccine prior to study onset. Descriptive characteristics are reported in Table 1.

Table 2 presents the Pearson correlations. Correlation analysis indicated a significant positive association between biological sex and testosterone (*r* = 0.82, *p* < 0.001). Biological sex was not significantly related to other study variables. 17β-estradiol was significantly positively associated with testosterone (*r* = 0.35, *p* = 0.002) and SARS-CoV-2 antibody persistence (*r* = 0.55, *p* < 0.001).

Table 3 and Table 4 present the hierarchical regression results. The first step of the regression analysis included biological sex as a predictor. The model did not significantly predict antibody persistence (*R*^2^ = 0.00, Adj. *R*^2^ = −0.01, F(1, 73) = 0.014, *p* = 0.91). Biological sex was not a significant predictor (*β* = −0.01, *p* = 0.91). The testosterone and 17β-estradiol hormones (zTest and zEstr) were added to the model in the second step. The addition of the hormones significantly improved model fit (Δ*R*^2^ = 0.34, F change(2, 71) = 18.64, *p* < 0.001) and significantly predicted antibody persistence (*R*^2^ = 0.34, Adj. *R*^2^ = 0.32, F(3, 71) = 12.436, *p* < 0.001). Biological sex (*β* = 0.043, *p* = 0.800) and testosterone (*β* = −0.24, *p* = 0.171) did not significantly contribute to the prediction, while 17β-estradiol emerged as a highly significant predictor of antibody persistence (*β* = 0.63, *p* < 0.001).

In step 3, interaction terms representing Sex × Estradiol and Sex × Testosterone were added as an exploratory step to examine whether associations between hormone concentrations and antibody persistence differed by biological sex. The addition of the interaction terms did not significantly improve model fit (Δ*R*^2^ = 0.04, F change(2, 69) = 2.06, *p* = 0.136). Examination of individual predictors indicated no significant Sex × Testosterone interaction (*β* = 0.55, *p* = 0.06) or Sex × Estradiol interaction (*β* = −0.15, *p* = 0.296). These findings suggest that the association between testosterone concentration and antibody persistence slightly differed between males and females, whereas the association between 17β-estradiol and antibody persistence remained consistent between males and females.

## 4. Discussion

The present study examined whether circulating sex hormone concentrations explained variation in SARS-CoV-2 antibody persistence, operationalized in weeks elapsed between SARS-CoV-2 infection and antibody assessment, beyond biological sex. The 17β-estradiol hormone significantly positively predicted SARS-CoV-2 antibody persistence, whereas biological sex and testosterone hormone were not significantly associated with antibody persistence. Furthermore, exploratory interaction analyses indicated that these associations were consistent across males and females, suggesting that endogenous hormone concentrations may provide a more informative biological framework for understanding variation in long-term humoral immunity than sex alone.

Our findings support the persistence of detectable SARS-CoV-2 anti-N IgG antibodies among healthy, college-aged adults following natural infection, with antibodies remaining detectable at an average of 15 weeks post-infection in both males and females. Previous research findings have noted age-related changes in SARS-CoV-2 antibody response and durability, often indicating less robust antibody memory in young adults compared to older adults [26]. In contrast, other studies have noted robust antibody responses following more severe SARS-CoV-2 cases, particularly in males [27]. While an association between disease severity and a more robust antibody response has been established, our present findings suggest that circulating sex hormones, particularly 17β-estradiol, may represent a more direct biological association of long-term antibody persistence than biological sex.

Biological sex is frequently used as a proxy for underlying physiological mechanisms despite substantial variability in hormone concentrations within both males and females. Endogenous sex hormones represent one biological factor that may contribute to variability in antibody persistence given evidence to influence both innate and adaptive immune function [28]. The positive association between salivary 17β-estradiol and SARS-CoV-2 antibody persistence observed in the present study may reflect the complex regulation of sex steroid metabolism rather than biological sex alone. Female participants were sampled during the early follicular phase, when estradiol concentrations are generally at their physiological nadir and may overlap with the normal physiological range observed in males [29,30]. Endogenous sex hormone concentrations remain influenced by multiple biological and lifestyle factors, which were not fully characterized in this cohort. Although males demonstrated a slightly higher mean salivary 17β-estradiol concentration than females in the descriptive statistics, this difference was not statistically significant. Accordingly, descriptive differences between males and females should be interpreted cautiously.

Males and females synthesize both testosterone and estradiol in varying concentrations, converting testosterone to estrogen through the enzyme estrogen synthase (aromatase). Aromatase activity is influenced by factors including adiposity, age, and alcohol consumption. This may account for the correlation between estradiol and testosterone, as well as estradiol and SARS-CoV-2 antibody persistence found in the present study.

Previous studies have demonstrated that body composition can alter estradiol concentrations in both sexes through changes in aromatase activity [31,32,33], while chronic alcohol consumption may further modify sex hormone balance through increased aromatization and disruption of hypothalamic–pituitary–gonadal axis signaling [34,35,36]. Although these variables were not measured in the present study, they represent sources of variability that may have influenced the salivary hormone concentrations observed among participants. Salivary estradiol primarily reflects the biologically active free fraction of circulating hormone [37], and thus distinctions between total and free estradiol should also be considered when comparing findings across studies. Collectively, these mechanisms may contribute to interindividual variability in endogenous sex hormone concentrations. These findings highlight the importance of considering endogenous hormone regulation and its physiological determinants in studies of antibody persistence, as hormone concentrations may better explain individual variability in humoral immune responses than categorization by biological sex alone.

The observed association between endogenous sex hormones and SARS-CoV-2 antibody persistence is biologically plausible given the well-established immunomodulatory effects of testosterone and estradiol. The following evidence provides a mechanistic framework for interpreting the present findings. Testosterone has been associated with modulation of both innate and adaptive immune processes wherein normal testosterone levels suppress pro-inflammatory cytokine production and inflammatory signaling pathways [38,39,40]. Estradiol has been shown to enhance several aspects of immune function, including B-cell activation, antibody production, and antiviral responses [13,15,41]. These immunomodulatory effects have led researchers to propose that sex hormones may contribute to observed differences in COVID-19 susceptibility, severity, and recovery.

17β-estradiol, the predominant estrogen during the reproductive years, plays a critical role in reproductive physiology while also influencing immune regulation [13,41,42]. Endogenous estradiol concentrations are generally highest during reproductive years and have been associated with enhanced antiviral immune responses and more efficient viral clearance [42]. Several additional pathways may explain the relationship between 17β-estradiol and antibody persistence. Estradiol has been associated with lower concentrations of pro-inflammatory cytokines and enhanced immune regulation, creating conditions that may support effective adaptive immune responses [42,43,44]. At the cellular level, estradiol has been shown to increase lysosomal activity, enhance catabolic function, and promote phagocytosis, mechanisms that may facilitate viral clearance during acute infection and subsequent immune memory [42,43]. These effects are mediated primarily through estrogen receptor alpha (ERα) and estrogen receptor beta (ERß), both of which regulate transcriptional pathways involved in innate and adaptive immune responses [43].

The observed association between 17β-estradiol and SARS-CoV-2 antibody persistence is biologically plausible and consistent with established literature describing the immunomodulatory effects of estrogen. Beyond SARS-CoV-2 infection, 17β-estradiol plays a central role in regulating numerous physiological processes, including reproduction, inflammation, vascular function, fibrosis, metabolism, neurological disorders, and antiviral immune responses [42]. Estradiol has long been recognized as an important regulator of B-cell development and function, promoting immunoglobulin (Ig) production and enhancing humoral immunity [45]. Such mechanisms may contribute to the maintenance of long-term antibody responses observed in the present study.

Furthermore, several studies have reported that females exhibit stronger and more durable humoral immune responses than males, including greater persistence of IgG antibodies following viral infection [41,42,44]. The adaptive immune response plays a critical role in viral clearance and long-term protection following SARS-CoV-2 infection. Activation of B lymphocytes leads to production of antibodies, initially dominated by immunoglobulin M (IgM) and subsequently characterized by Class-Switch Recombination to immunoglobulin G (IgG) with assistance from physical and chemical signals of CD4+ T-cells [9,46]. Although antibody concentrations generally decline over time, memory B-cells and T-cells may persist and contribute to protection upon re-exposure to the virus [3,10]. However, considerable variability exists in the magnitude and durability of antibody response, with factors such as disease severity, viral variant, and biological sex influencing antibody persistence [2,27,47].

The pluralistic etiology of sex-related effects on immune response stems from sex-encoded genes on the X or Y chromosome and sex-receptor signaling that influence genetic expression in immune cell function [16]. Concentrations of sex hormones change throughout life and cyclically fluctuate in menstruating women. Since sex hormones modulate various immune functions, the extent of antibody production and antibody durability may also fluctuate with sex hormone concentration, age, and severity of infection. Study findings contribute to a growing body of literature examining interactions between endocrine and immune pathways. Collectively, these findings suggest that endogenous sex hormones, particularly 17β-estradiol, may contribute to individual variation in immunologic response.

### Limitations

Findings of the cross-sectional study will not establish causal inference regarding relationships between endogenous sex hormone concentrations and SARS-CoV-2 antibody persistence. Although significant associations may provide insight into potential biological mechanisms, longitudinal studies are necessary to determine whether hormone concentrations directly influence post-infection immunity over time. Participants were recruited from one college campus, resulting in a relatively small sample size, which may limit the generalizability of the findings to broader populations. The relatively young and healthy characteristics of the sample may differ from clinical or older adult populations that experience distinct immune and endocrine responses. Hormone concentrations were measured at a single time point. Although efforts were made to minimize biological variability by standardizing collection procedures and coordinating all female sampling, circulating concentrations of 17β-estradiol and testosterone exhibit temporal fluctuations that may not be fully captured by a single assessment. Physiological and lifestyle factors known to influence aromatase activity and endogenous sex hormone concentrations were not assessed. Consequently, unmeasured variation in these characteristics may have contributed to differences in salivary estradiol and testosterone concentrations observed among participants. Future studies should incorporate repeated hormone measurements and account for biological and lifestyle factors that influence endogenous hormone concentrations to further elucidate endocrine contributions to humoral immunity.

Antibody persistence was estimated using the number of weeks elapsed since participants’ self-reported positive SARS-CoV-2 test. Although this approach provided a practical measure of time since infection, it is subject to potential recall error and does not account for undocumented reinfections that may have influenced antibody status. Finally, SARS-CoV-2 antibodies were confirmed using a qualitative rapid immunoassay that determined the presence or absence of nucleocapsid-specific IgG antibodies. Consequently, the study was unable to evaluate antibody titers or characterize the magnitude of humoral immune responses. Further investigations incorporating quantitative serological assays may provide greater knowledge regarding relationships between hormone concentrations and antibody kinetics. Future longitudinal studies are needed to characterize individual changes in antibody persistence over time. Despite these limitations, the study contributes additional evidence regarding the role of sex hormones, particularly 17β-estradiol, in shaping post-infection immune responses.

## 5. Conclusions

Sex classification is often used to examine differences in disparities of SARS-CoV-2 viral severity and immune response. The present study indicates the multifactorial nature of endocrine–immune function and the strong association between 17β-estradiol and antibody persistence, operationalized as weeks elapsed from SARS-CoV-2 infection until antibody assessment. Study findings indicate that individual variation in sex hormones provides biological explanation for differences in SARS-CoV-2 antibody persistence beyond biological sex but is limited by study design and subjective reporting of viral infection history. Our analysis provides a foundation for future investigations into the role of endogenous sex hormones in SARS-CoV-2 humoral immunity and supports the need to evaluate hormone regulation alongside biological sex when characterizing immune responses.

## Figures and Tables

**Table 1:** Descriptive statistics for main study variables.

	Mean	Standard Deviation	Skewness	Kurtosis
17β-Estradiol pg/mL				
Total (N = 75)	1.52	0.65	0.03	−0.91
Female (N = 47)	1.40	0.57	0.22	−0.34
Male (N = 28)	1.70	0.73	−0.46	−1.06
Testosterone pg/mL				
Total (N = 75)	89.05	57.61	0.95	−0.15
Female (N = 47)	52.94	20.55	−0.25	−1.05
Male (N = 28)	149.68	47.88	−0.02	−1.23
Antibody persistence ^a^				
Total (N = 75)	15.41	7.13	0.37	−0.63
Female (N = 47)	15.49	7.55	0.40	−0.69
Male (N = 28)	15.29	6.49	0.26	−0.57

^a^ SARS-CoV-2 antibody longevity measured in weeks since infection.

**Table 2:** Correlation matrix.

	1.	2.	3.	4.
1. Biological sex ^a^	—			
2. 17β-Estradiol pg/mL	0.224	—		
3. Testosterone pg/mL	0.818 **	0.350 **	—	
4. Antibody persistence ^b^	−0.014	0.554 **	0.014	—

** Correlation is significant at the 0.01 level (2-tailed). Note: Sample size (N = 75). ^a^ Biological sex recorded (0 = female, 1 = male). ^b^ SARS-CoV-2 antibody persistence measured in weeks elapsed since infection until antibody assessment.

**Table 3:** Hierarchical regression model summary with SARS-CoV-2 antibody persistence (weeks elapsed since infection) as DV.

	*R*	*R* ^2^	Adjusted *R*^2^	*SE* (Est.)	Change Statistics				
					Δ*R*^2^	F Change	df1	df2	*p*
1	0.014	0.000	−0.014	7.18013	0.000	0.014	1	73	0.906
2	0.587	0.344	0.317	5.89532	0.344	18.643	2	71	<0.001
3	0.618	0.381	0.336	5.80963	0.037	2.055	2	69	0.136

Note: Model 1 IVs: Biological sex. Model 2 IVs: Biological sex, z-score: 17β-estradiol, z-score: testosterone. Model 3 IVs: Biological sex, z-score: 17β-estradiol, z-score: testosterone, SexEstr, SexTest.

**Table 4:** Hierarchical multiple regression with SARS-CoV-2 antibody persistence (weeks elapsed since infection) as DV.

Step and Independent Variables (IVs)		*B*	*SE*	*β*	*t*	*p*
1	Biological Sex	−0.204	1.714	−0.014	−0.119	0.906
2	Biological Sex	0.628	2.462	0.043	0.255	0.800
	17β-Estradiol	4.486	0.737	0.629	6.089	**<0.001**
	Testosterone	−1.724	1.247	−0.242	−1.382	0.171
3	Biological Sex	1.462	2.509	0.100	0.583	0.562
	17β-Estradiol	5.117	0.986	0.717	5.191	**<0.001**
	Testosterone	−5.564	2.424	−0.780	−2.295	0.025
	Sex × Estradiol	−1.537	1.460	−0.151	−1.053	0.296
	Sex × Testosterone	5.480	2.836	0.551	1.932	0.057

Note: *B* = unstandardized coefficient; *SE* = standard error of B; *β* = standardized coefficient; DV = dependent variable; IV = independent variable. Bold *p*-values highlight IVs with *p* < 0.05 in that step.

## Data Availability

The data presented in this study are available on request from the corresponding author due to privacy concerns involving the college-aged participants.

## Abbreviations

The following abbreviations are used in this manuscript:

SARS-CoV-2	Severe Acute Respiratory Syndrome Coronavirus 2
COVID-19	Coronavirus Disease of 2019
IgM	Immunoglobulin M
IgA	Immunoglobulin A
IgG	Immunoglobulin G
